# Alterations in Metabolism and Diurnal Rhythms following Bilateral Surgical Removal of the Superior Cervical Ganglia in Rats

**DOI:** 10.3389/fendo.2017.00370

**Published:** 2018-01-09

**Authors:** Malena L. Mul Fedele, Maria D. Galiana, Diego A. Golombek, Estela M. Muñoz, Santiago A. Plano

**Affiliations:** ^1^Science and Technology, Universidad Nacional de Quilmes (UNQ), Bernal, Argentina; ^2^Institute of Histology and Embryology of Mendoza (IHEM—CONICET), Universidad Nacional de Cuyo, Mendoza, Argentina; ^3^Chronophysiology Laboratory, Institute for Biomedical Research (BIOMED—CONICET), UCA Pontificia Universidad Católica Argentina, Buenos Aires, Argentina

**Keywords:** superior cervical ganglion, SCGx, circadian rhythm, metabolism, melatonin

## Abstract

Mammalian circadian rhythms are controlled by a master pacemaker located in the suprachiasmatic nuclei (SCN), which is synchronized to the environment by photic and nonphotic stimuli. One of the main functions of the SCN is to regulate peripheral oscillators to set temporal variations in the homeostatic control of physiology and metabolism. In this sense, the SCN coordinate the activity/rest and feeding/fasting rhythms setting the timing of food intake, energy expenditure, thermogenesis, and active and basal metabolism. One of the major time cues to the periphery is the nocturnal melatonin, which is synthesized and secreted by the pineal gland. Under SCN control, arylalkylamine *N*-acetyltransferase (AA-NAT)—the main enzyme regulating melatonin synthesis in vertebrates—is activated at night by sympathetic innervation that includes the superior cervical ganglia (SCG). Bilateral surgical removal of the superior cervical ganglia (SCGx) is considered a reliable procedure to completely prevent the nocturnal AA-NAT activation, irreversibly suppressing melatonin rhythmicity. In the present work, we studied the effects of SCGx on rat metabolic parameters and diurnal rhythms of feeding and locomotor activity. We found a significant difference between SCGx and sham-operated rats in metabolic variables such as an increased body weight/food intake ratio, increased adipose tissue, and decreased glycemia with a normal glucose tolerance. An analysis of locomotor activity and feeding rhythms showed an increased daytime (lights on) activity (including food consumption) in the SCGx group. These alterations suggest that superior cervical ganglia-related feedback mechanisms play a role in SCN-periphery phase coordination and that SCGx is a valid model without brain-invasive surgery to explore how sympathetic innervation affects daily (24 h) patterns of activity, food consumption and, ultimately, its role in metabolism homeostasis.

## Introduction

The circadian system, a set of biological clocks that regulate almost all physiological and behavioral processes, has evolved to adapt the organism’s physiology to cyclic environmental changes (1–4). In mammals, the master clock resides in the suprachiasmatic nuclei (SCN) of the hypothalamus and is mainly synchronized by the light–dark (LD) cycle (5). The circadian system also includes peripheral clocks, entrained by the SCN *via* neural and humoral cues, such as rhythmically secreted hormones (6–8), and other SCN-independent cues like food (9).

One of the major physiological processes controlled by the SCN is metabolism, including metabolic rate and circadian rhythms of food intake (3). Food consumption is normally confined to the wake/active phase, while fasting periods occur during the rest/sleep phase, correlating to the anabolic, and catabolic phases of metabolism, respectively (10). Alterations of the circadian pacemaker can lead to metabolic pathologies, such as obesity or metabolic syndrome (11). For example, shift work, chronic forced circadian desynchronization or mutations of clock genes can affect the pattern of food intake and lead to increased levels of circulating triglycerides, and adipose tissue masses resulting in an augmented body weight (12–15).

Melatonin is a hormone produced by the pineal gland during the dark phase and is considered one of the most important circadian outputs (16). It regulates major physiological processes, including the sleep–wake cycle, and lipid and glucose metabolism (17–22). The SCN interact with the pineal gland through the sympathetic neurons of the superior cervical ganglia (SCG) (23). This interaction modulates the arylalkylamine *N*-acetyltransferase (AA-NAT) activity, the main enzyme responsible for melatonin rhythm generation in vertebrates (24). The elimination of the pineal melatonin rhythm, or a reduction of its amplitude, renders the circadian pacemaker a less self-sustained, often damped, oscillatory system (25). On the other hand, forced circadian desynchronization induced by an LD cycle of 22 h in rats (26) or by shift work in humans (27) disrupts rhythmic melatonin secretion.

The SCG are the uppermost ganglia of the paraventral sympathetic chain and innervate the pineal gland, among others structures (28). Superior cervical ganglionectomy (SCGx) is a reliable model to study the role of sympathetic innervation on neuroendocrine interactions (29–31). Moreover, SCGx has been used to determine the influences of the circadian clock (i.e., the SCN) on neuroendocrine functions. In this sense, SCGx disrupts the circadian system by depressing melatonin secretion and suppressing its rhythm (32, 33), presumably by the inhibition of pineal AA-NAT activity (34). This also results in an abolition of the rhythmic excretion of urinary 6-sulphatoxymelatonin, a melatonin metabolite (35). In addition, the SCG also cover other territories such as other glands, brain areas, and the cardiovascular system, which might also be implied in metabolic regulation (36–41).

Taking into account that the lack of melatonin can produce circadian alterations, and that sympathetic innervation from the SCG covers diverse neuroendocrine effectors, the aim of our work was to study if SCGx can affect rat metabolism and whether this is related to an impairment of the circadian clock.

## Materials and Methods

### Ethics Statement

All animal procedures were approved by the Institutional Animal Care and Use Committee at the School of Medicine, National University of Cuyo, Mendoza, Argentina (Protocol ID 9/2012) and were conducted in accordance with the National Institutes of Health’s Guide for Care and Use of Laboratory Animals and the Animal Research: Reporting *In Vivo* Experiments (ARRIVE) Guidelines.

### Animals

Young (3 months old) male Wistar rats were raised in our colony and maintained in a 12:12 h LD cycle (with zeitgeber time 12—ZT 12—defined as the time of lights off; light intensity averaging 300 lux at the cage level), in a controlled environment with food and water *ad libitum*.

### Locomotor Activity Rhythms

Animals were transferred to individual cages equipped with infrared motion sensors. Locomotor activity was assessed by the interruption of the infrared beam and recorded every 5 min (Archron, Argentina). The locomotor activity rhythm analysis was performed using the “el Temps” program (http://www.el-temps.com). Locomotor activity onset was defined as the 10-min bin that contained at least 50% of the maximum activity/bin followed by another bin of at least another 50% of the maximum activity bin within 40 min. Entrainment to the LD cycle was confirmed by periodogram analysis (χ^2^ test). Phase angle was measured as the difference (in minutes) between activity onset and lights off. Total daytime activity was assessed by the area under the curve (AUC) of the waveform of each animal. Activity was expressed as a percentage of the total activity or relative activity by comparing post-surgery activity to the activity counts of the 3 weeks previous to the surgery (pre-surgery) as the post-/pre-ratio.

### Surgery

Bilateral superior cervical ganglionectomy (SCGx) was performed as described by Savastano et al. (31). Briefly, under ketamine (50 mg/kg of body weight)/xylazine (5 mg/kg of body weight) anesthesia, the ventral neck region was shaved and disinfected. The salivary glands were exposed through a 2.5 cm vertical incision and retracted to uncover the underlying muscles. The carotid bifurcations were identified through the carotid triangles and the SCG were removed after sectioning the sympathetic trunks, the external carotid nerves, and the internal carotid nerves. For sham-operated animals, the same procedure was performed but the ganglia were not removed.

### Animal Weight and Food Intake Measurements

Body weight and food consumption were monitored weekly at ZT10. After a 3-week pre-surgery baseline, animals were subjected to bilateral SCGx or a sham procedure (*n* = 9 per group), and body weight and food intake were measured for another 10 weeks. Food efficiency (FE) was analyzed by the body weight/food intake ratio.

The food intake rhythm was analyzed in both groups at week 11. Daytime (i.e., during lights on) and nighttime (during lights off) food intakes were measured daily at the end of the light and dark phases for 10 days (*n* = 5 per group). Daytime and nighttime feedings were expressed as a percentage of total food consumed per day.

### Glycemia and Glucose Tolerance Test (GTT)

At week 10, glycemia was measured at ZT10 using PTS PanelsTM test strips for CardioChekTM Brand Analyzer (Hannover, Germany) (*n* = 9 per group).

At week 13, a GTT was performed after 18 h fast (*n* = 5 per group). Glycemia was measured as mentioned above before and 15, 30, 60, and 120 min after glucose administration (orogastric, 3 g/kg of body weight from a 30% solution of d-glucose), at ZT10. The AUC of glycemia vs. time was calculated above each individual baseline (basal glycemia).

### Fat Weight Measurements

At the end of week 13, animals were decapitated under anesthesia, and epididymal, retroperitoneal, mesenteric, and inguinal adipose tissues were collected and weighed (*n* = 5 per group). Fat weight was expressed as relative to body weight.

### Statistical Analysis

Data were expressed as mean ± SEM and analyzed using PRISM5 (GraphPad Software Inc., La Jolla, CA, USA). Statistical difference between means was determined by Student’s *t*-test. For the grouped statistical analysis, two-way ANOVA or repeated measures two-way ANOVA was used with Bonferroni as post-test. *p* < 0.05 was considered significant and *p* < 0.01 highly significant.

## Results

### Global Metabolism Is Affected by Bilateral Superior Cervical Ganglionectomy

**Figure 1:**
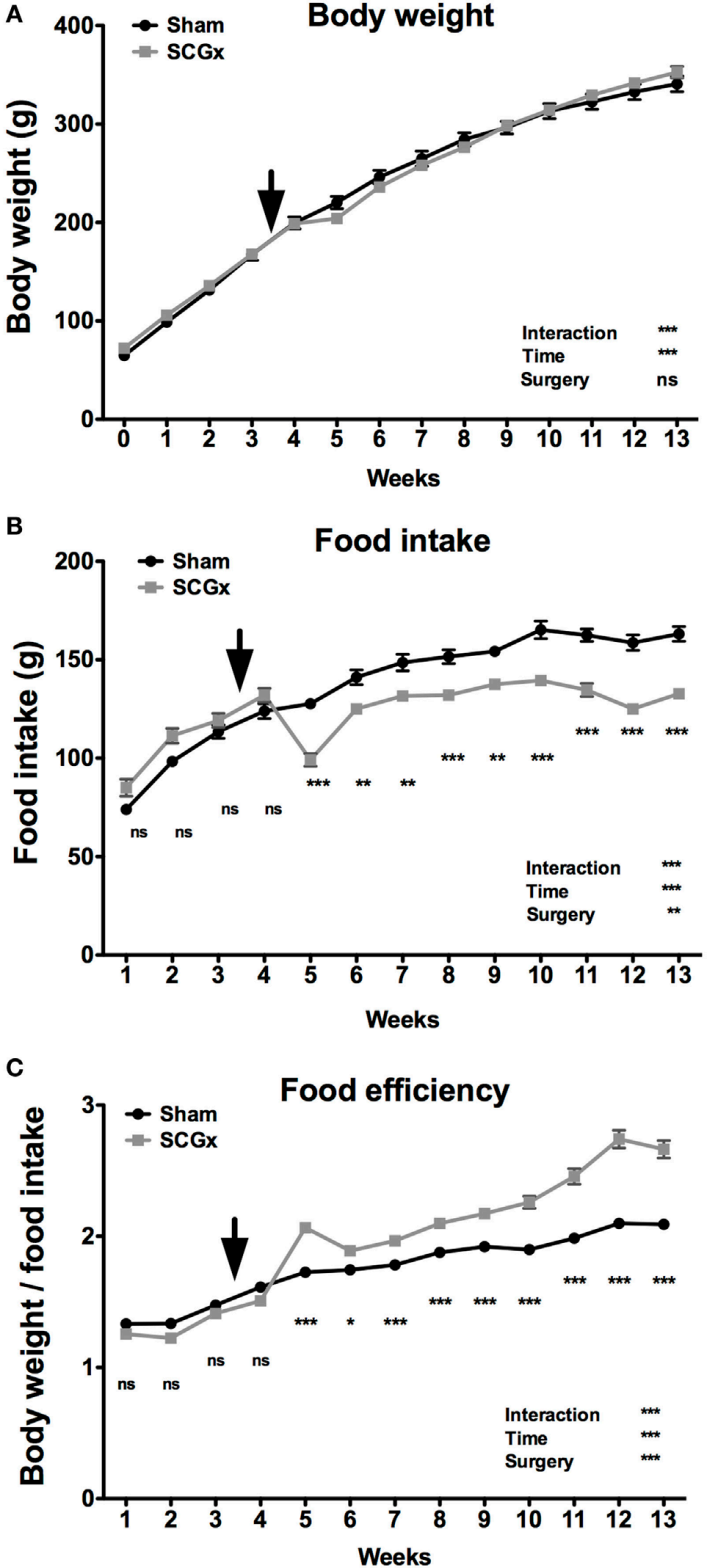
Bilateral superior cervical ganglionectomy affects metabolic variables. Rats subjected to SCGx at week 3.5 did not exhibit differences in body weight [**(A)**; repeated measures two-way ANOVA: *p* = 0.0002, *F* = 3.229 for interaction, *p* < 0.0001, *F* = 1,588 for time, *p* > 0.05, *F* = 0.008 for surgery; *n* = 9 per group], but had significant lower food intake throughout the 10 weeks after surgery [**(B)**; repeated measures two-way ANOVA: *p* < 0.0001, *F* = 35.51 for interaction, *p* < 0.0001, *F* = 222.8 for time, *p* = 0.0015, *F* = 14.92 for surgery, followed by Bonferroni post-tests: ***p < 0.001, **p < 0.01; *n* = 9 per group]. A food efficiency (body weight/food intake ratio) analysis demonstrated metabolic differences between the two groups with higher levels in ganglionectomized animals [**(C)**; repeated measures two-way ANOVA: *p* < 0.0001, *F* = 42.75 for interaction, *p* < 0.0001, *F* = 374.7 for time, *p* < 0.0001, *F* = 76.49 for surgery, followed by Bonferroni post-tests: ***p < 0.001, *p < 0.05; *n* = 9 per group]. The rats used in this work were still growing from young-to-adulthood and therefore increasing their body mass and food consumption over time. Arrows indicate the day of surgery. Repeated measures two-way ANOVA results are depicted at the bottom right of each figure. Values are given as mean ± SEM.

To study the effect of SCGx on rat metabolism, animals were subjected to ganglionectomy or a sham procedure at the middle of week 3 (*n* = 9 per group). Body weight and food consumption were measured, and FE (body weight/food intake ratio) was calculated. Rats subjected to SCGx did not exhibit differences in body weight (Figure 1A) but had significant lower food intake when compared with sham animals (Figure 1B), throughout the 10 weeks after surgery. An FE analysis (42) showed metabolic differences between the two groups. FE was higher in ganglionectomized animals, revealing that these rats gained more body mass per gram of consumed food than controls (Figure 1C).

### Ganglionectomy Increases Daytime Locomotor Activity

**Figure 2:**
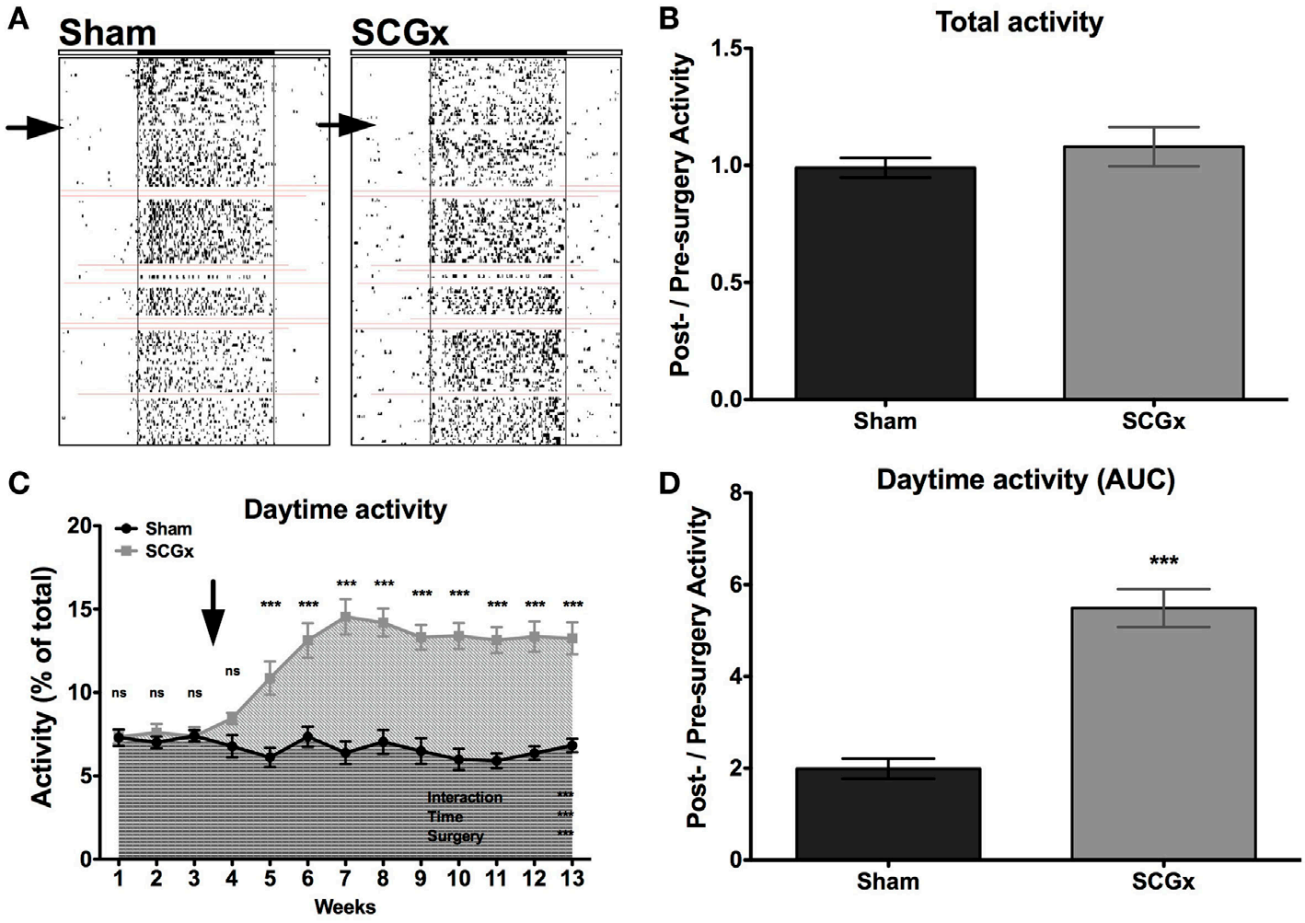
Ganglionectomy affects locomotor activity rhythm. **(A)** Representative actograms for animals subjected to SCGx or sham procedure (*n* = 9 per group). Red lines indicate the moments that the system did not record activity. **(B)** A locomotor activity analysis showed no differences in the levels of total activity, as post-surgery/previous-to surgery ratio (SCGx group, 1.08 ± 0.083; sham group, 0.99 ± 0.042; values are given as mean ± SEM; *t*-test: *p* < 0.353; *n* = 9 per group), but the activity of SCGx animals during daytime (i.e., during lights on) increased after surgery and remained higher throughout the 10-week post-surgery interval [**(C)**; repeated measures two-way ANOVA: *p* < 0.0001, *F* = 16.55 for interaction, *p* < 0.0001, *F* = 11.50 for time, *p* < 0.0001, *F* = 43.69 for surgery, followed by Bonferroni post-tests: ***p < 0.001; *n* = 9 per group]. This increased daytime activity is evidenced in the area under the curve (AUC) from post-surgery/pre-surgery ratio, that was significantly higher in the SCGx animals when compared with the sham group [**(D)**; SCGx group: 5.492 ± 0.4126; sham group: 1.992 ± 0.2212; values are given as mean ± SEM; *t*-test: ***p < 0.0001, *t* = 7.475; *n* = 9 per group]. Repeated measures two-way ANOVA results are expressed at the bottom right of the figure. Asterisks above the curve indicate significant *p*-values of the Bonferroni post-test. The arrows correspond to the day of surgery.

Rats subjected to SCGx or sham surgeries (*n* = 9 per group) were placed individually in cages with infrared sensors to study their activity distribution during the day. An activity rhythm analysis demonstrated that entrainment to the LD cycle and activity phase angle were not affected by ganglionectomy (Table 1; Figure 2A). Moreover, SCGx animals did not show differences in the levels of total activity as post-/pre-surgery ratio (Table 1; Figure 2B; SCGx group: 1.08 ± 0.083; sham-operated group: 0.99 ± 0.042; data expressed as mean of post-/pre-surgery ± SEM). However, locomotor activity of ganglionectomized animals during the lights-on phase increased after surgery and remained higher throughout the 10-week post-surgery interval (Figure 2C). Moreover, the relation between the AUC of daytime activity after and before surgery was significantly higher in the SCGx animals (Table 1; Figure 2D; SCGx group: 5.492 ± 0.4126; sham group: 1.992 ± 0.2212; data expressed as mean of post-/pre-surgery ± SEM). This increase occurs at the expense of a reduced nighttime activity (Table 1, SCGx group: 0.91 ± 0.005; sham-operated group: 1.01 ± 0.003; data expressed as mean of post-/pre-surgery ± SEM).

**Table 1:** Effects of SCGx on the diurnal rhythm of locomotor activity.

	Sham	SCGx	*p*-Value
Period (min)	1,441 ± 0.645	1,442 ± 1.323	0.522
Phase angle (min)	6.50 ± 1.190	7.00 ± 1.080	0.766
Total activity (post-/pre-surgery)	0.99 ± 0.042	1.08 ± 0.083	0.351
Daytime activity (post-/pre-surgery)	1.99 ± 0.221	5.49 ± 0.412	<0.0001
Nighttime activity (post-/pre-surgery)	1.01 ± 0.003	0.91 ± 0.005	<0.0001

### Ganglionectomy Increases Food Intake during Daytime

**Figure 3:**
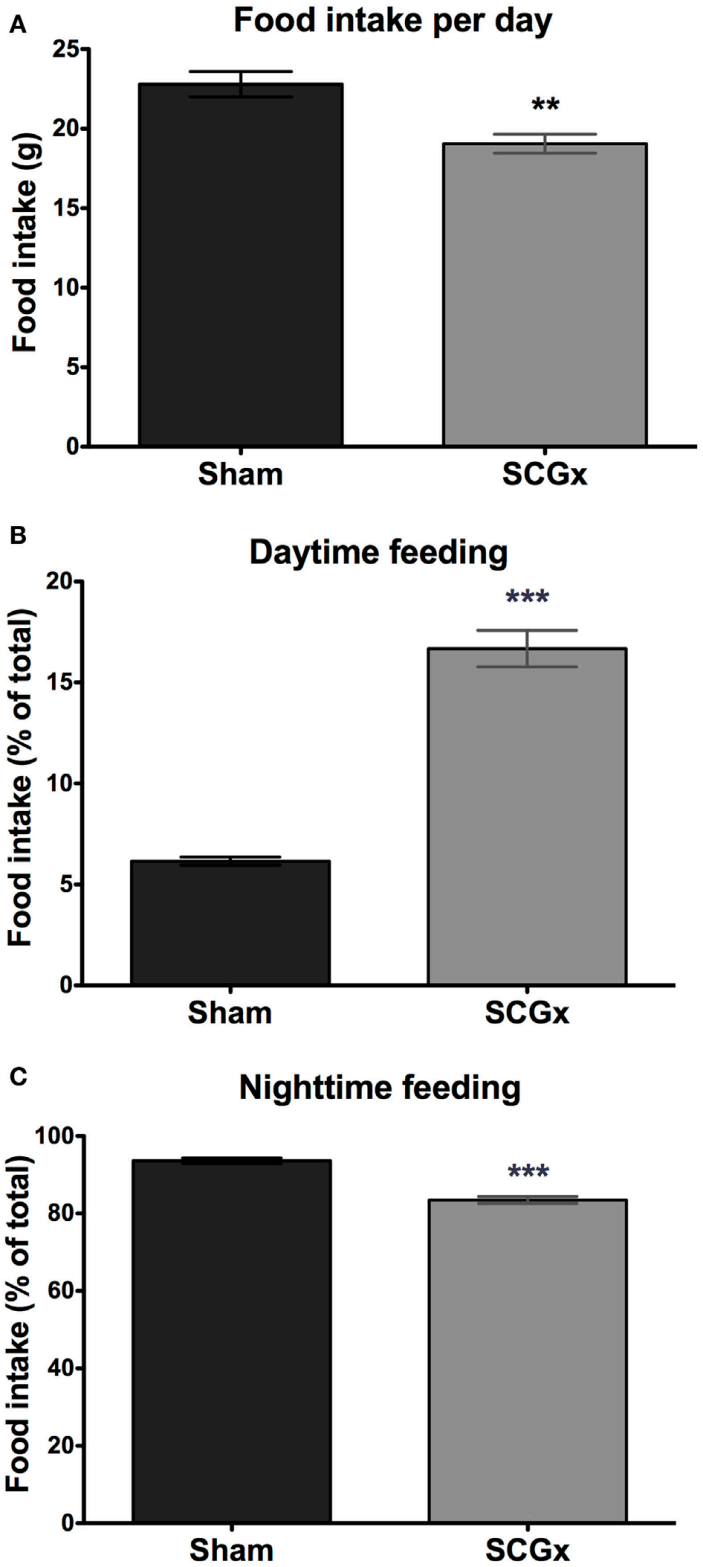
Daytime feeding is increased in SCGx animals. Ganglionectomized animals had a lower level of food intake [**(A)**; SCGx group: 19.06 ± 0.5960 g; sham group: 22.80 ± 0.8027 g; values are given as: mean ± SEM; *t*-test: **p = 0.0057, *t* = 3.738; *n* = 5 per group]. Feeding rhythm was also affected: daytime (i.e., during lights on) food consumption was higher in SCGx rats [**(B)**; SCGx group: 16.68 ± 0.9030 g; sham group: 6.160 ± 0.2015 g; *t*-test: ***p < 0.0001, *t* = 11.37; *n* = 5 per group], and lower during the nighttime (i.e., during lights off) [**(C)**; SCGx group: 83.48 ± 0.8864 g; sham group: 93.63 ± 0.7122 g; *t*-test: ***p = 0.0001, *t* = 8.926; *n* = 5 per group], compared with sham animals. Values are given as mean ± SEM.

We next studied the daily pattern of food consumption, which can be affected by circadian alterations (13). Ganglionectomized animals had a lower level of food intake per day (Figure 3A; 19.06 ± 0.5960 g for SCGx group; 22.80 ± 0.8027 g for sham group, *n* = 5 per group).

As it was observed with the activity rhythm, a food intake rhythm analysis revealed increased food consumption during daytime (Figure 3B; 16.68 ± 0.9030 g for SCGx group; 6.160 ± 0.2015 g for sham group), and a slightly but significantly lower feeding activity during the night (Figure 3C; 83.48 ± 0.8864 g for SCGx group; 93.63 ± 0.7122 g for sham group).

### SCGx Animals Exhibit Lower Basal Levels of Blood Glucose but Higher Adipose Tissue

**Figure 4:**
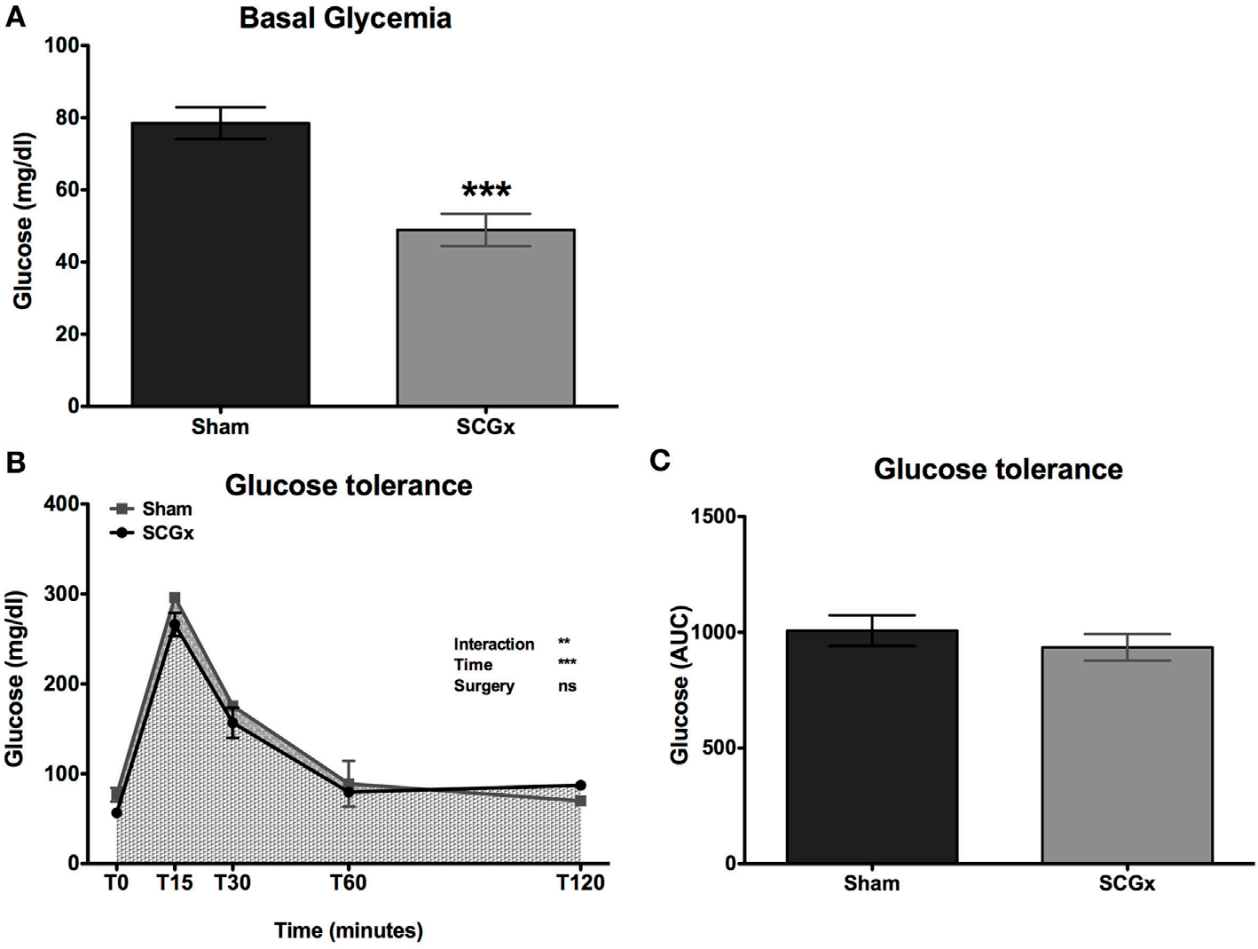
SCGx animals exhibit lower basal levels of blood glucose, with normal glucose tolerance. Basal glucose levels at ZT10 were measured at week 10. We found lower levels in SCGx rats when compared with the sham ones [**(A)**; SCGx group: 48.89 ± 4.464 mg/dl; sham group: 78.50 ± 4.392 mg/dl; *t*-test: ***p = 0.0003, *t* = 4.706; *n* = 9 per group]. At week 13, a glucose tolerance test (GTT) was performed (*n* = 5 per group). Glycemia was measured before and 15, 30, 60, and 120 min after glucose administration. There were no differences in glycemia kinetics **(B)** or in the area under the curve of the GTT [**(C)**; SCGx group: 935 ± 57.04 mg/dl; sham group: 1,008 ± 65.66 mg/dl; *t*-test: *p* = 0.214, *t* = 0.834; *n* = 5 per group] between ganglionectomized and sham animals. Values are given as: mean ± SEM. Repeated measures two-way ANOVA results are shown at the right of the figure.

Six weeks after surgery, a glycemia analysis at ZT10 showed lower levels of blood glucose in SCGx rats (Figure 4A; 48.89 ± 4.464 mg/dl for SCGx group; 78.50 ± 4.392 mg/dl for sham group; *n* = 9 per group).

At week 13, a GTT was performed (*n* = 5 per group). Surprisingly, there were no differences in glycemia kinetics (Figure 4B) or in the AUC of the GTT (Figure 4C; 935 ± 57.04 mg/dl for SCGx; 1,008 ± 65.66 mg/dl for sham) between ganglionectomized and sham animals.

**Figure 5:**
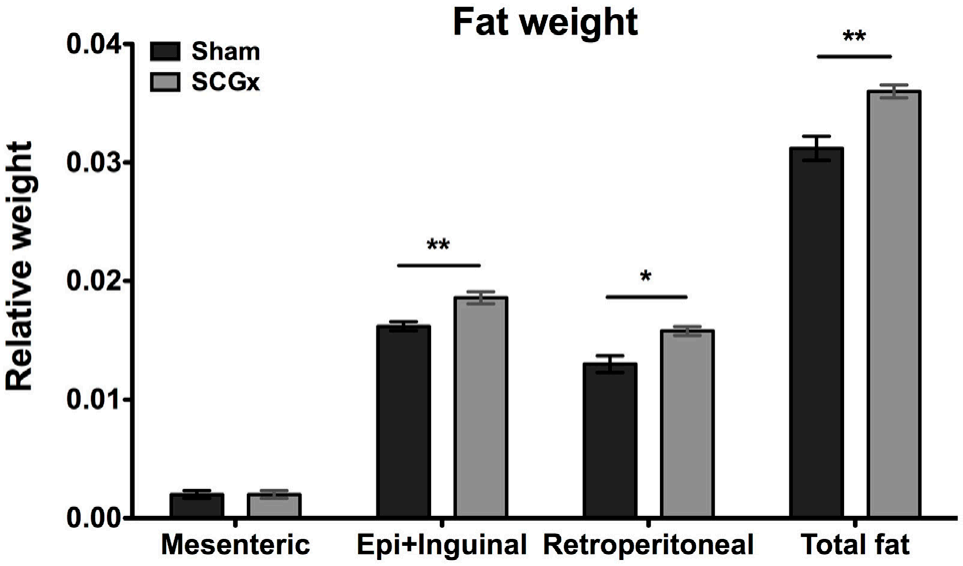
Ganglionectomized rats exhibit higher levels of adipose tissue. Epididymal + inguinal (Epi + Inguinal), retroperitoneal, and mesenteric fat were collected at the end of week 13, and their weights were relativized to body weight for each animal. Fat tissue was significantly increased in SCGx compared with sham animals (for epididymal + inguinal fat, SCGx group: 0.0186 ± 0.0005; sham group: 0.0162 ± 0.0004; *t*-test: **p = 0.0053, *t* = 3.795; for retroperitoneal fat, SCGx group: 0.0154 ± 0.0002; sham group: 0.0130 ± 0.0007071; *t*-test: *p = 0.0125, *t* = 3.207, for mesenteric fat, SCGx group: 0.002 ± 0.0003; sham group: 0.002 ± 0.0003; *t*-test: *p* = 1, *t* = 0; and for total fat, as the collective weight of epididymal + inguinal, retroperitoneal, and mesenteric fat, SCGx group: 0.0362 ± 0.0007; sham group: 0.0318 ± 0.0011; *t*-test: **p = 0.0032, *t* = 4.14; values are given as: mean ± SEM; *n* = 5 per group).

Finally, to better understand the increased body mass in SCGx animals, we studied the fraction of the body weight that is represented by adipose tissue. For this, we measured the levels of mesenteric, epididymal, retroperitoneal, and total fat at the end of week 13 (Figure 5), and found adipose tissue significantly increased in SCGx when compared with sham animals (epididymal fat: SCGx group, 0.0186 ± 0.0005; sham group, 0.0162 ± 0.0004; retroperitoneal fat: SCGx group, 0.0154 ± 0.0002; sham group, 0.0130 ± 0.0007; mesenteric fat: SCGx group, 0.002 ± 0.0003; sham group, 0.002 ± 0.0003; total fat: SCGx group, 0.0362 ± 0.0007; sham group, 0.0318 ± 0.0011; *n* = 5 per group).

## Discussion

The impact of the superior cervical ganglionectomy (SCGx) on hormone secretion, and blood glucose and insulin release has been reported before (40, 43–46) but its role on body weight homeostasis remains to be fully established. In this work, we assessed the impact of SCGx on rat metabolism and diurnal rhythms. Rats subjected to SCGx showed: (1) increased FE (i.e., gained more weight per gram of food consumed); (2) increased activity during the lights-on phase of the photoperiod; (3) increased feeding during daytime; (4) reduced glucose levels, without changes in glucose tolerance, at ZT10; and (5) increased adipose tissue mass.

The SCG provide sympathetic innervation to diverse areas including the hypothalamus, the pineal gland, cephalic blood vessels, the choroid plexus, the eye, the myocardium, the salivary and thyroid glands, and the carotid body (12, 40, 41). Removal of the superior cervical ganglia can cause loss of vasoconstriction control of brain and pituitary blood vessels (47), changes in cerebrospinal fluid production from the choroid plexus (48), and other central effects in response to partial sympathetic denervation (49). Moreover, abolition of the peripheral sympathetic innervation of the brain by SCGx is associated with several neuroendocrine changes in mammals, which include the disruption of water balance (37), and the alteration of normal photoperiodic control of reproduction (50, 51).

As previously mentioned, the mammalian circadian system is held in synchrony by the SCN through endocrine and autonomic outputs (52, 53). One of the mayor endocrine cues is the pineal hormone melatonin. Its synthesis and release is driven by the SCN through a multisynaptic pathway relaying in the SCG (54, 55). This interaction determines the rhythmic production of the hormone, whose day–night profile is modulated by daylength (23), encoding photoperiodic changes in the metabolic state (56).

Previous evidences have shown that SCGx decreases the secretion of melatonin and suppresses its rhythm (32, 33). The relationship between melatonin and the circadian control of metabolism has been demonstrated before. Pinealectomy and melatonin administration or replacement (57, 58) significantly changes body weight, as well as glucose levels and its utilization in different tissues (59). In our model, we found decreased levels of glucose at ZT10, but a GTT showed no differences between SCGx and sham-operated animals. In contrast, pineal ablation in rats was shown to increase glucose levels (57).

Furthermore, leptin secretion is strongly associated with glucose and lipid metabolism, and has been shown to be modulated by melatonin (60). Moreover, the administration of melatonin in experiments conducted in rats and rabbits induced a reduction in body weight, serum lipids, adiposity, blood glucose, and insulin levels associated with the intake of a high-fat diet, suggesting a protective role of melatonin (20, 61, 62).

Taking into account our results, SCGx mimics the effect of pinealectomy on the neuroendocrine system only in some aspects, affecting several areas that include, but are not restricted to, the pineal gland. Although we cannot state that all SCGx-induced changes presented here are exerted *via* a suppressed pineal function, it is tempting to speculate that the diurnal timing of locomotion and feeding might be related to the lack of melatonin feedback to the circadian clock.

The importance of timed feeding and circadian physiology of metabolism has been extensively studied (63, 64). In this sense, an increased fat anabolism during daytime (i.e., the rest phase) due to food consumption at this time, may explain the lower levels of blood glucose, and lead to increased adiposity in the SCGx group. Previous studies showed that animals fed during the light phase exhibit an increased body weight and food consumption, alterations in leptin, insulin, corticosterone, glucose, and free fatty acid levels in plasma, fat accumulation, liver steatosis, and metabolic syndrome (65–69). These alterations arise from a completely reversed clock-gene expression in the liver, kidney, heart, and pancreas, without affecting SCN function (9).

On the other hand, SCGx rats exhibit significantly augmented serum corticosterone and adreno-corticotropin hormone levels, and a suppression of their rhythm (35, 70). Glucocorticoids (GCs) can stimulate the *de novo* synthesis of lipids (71). It has been reported that rats exposed to long-term treatment with GCs show a slower body weight gain, reduced food intake, and increased epididymal fat mass (72). Some of the effects reported here might be related to alterations in GC turnover that, in turn, could lead to the increase in FE and lipid accumulation. Indeed, the role of the sympathetic neuro-adipose connections in the regulation of lipolysis and body weight has been studied before (73). Sympathetic denervation leads to an increase in adipose tissue, while nerve stimulation results in fatty acid release, and sympathetic or ganglionic blockade inhibits the mobilization of lipids (74–76). Leptin production is also under the control of the sympathetic system (77), with participation of the SCG (78).

Regarding light synchronization, it has been demonstrated that pinealectomy accelerates the re-entrainment of rats to the new LD schedule (79–82). Moreover, in rodents, melatonin administration synchronizes free-running rhythm and accelerates re-entrainment after phase shifts of the LD cycles (83–85), and reinforces entrainment to shortened 22 h LD cycles in both SCGx and pinealectomized rats (86). We studied the effect of SCGx on the entrainment to the LD cycle and found no significant differences on period, phase angle, or total locomotor activity between SCGx and sham-operated animals. However, SCGx rats showed significant differences in activity during daytime (lights on). In addition, food intake analysis evidenced augmented food consumption during daytime, which may correlate with the activity bouts under the light phase.

Also, it was previously observed that bilateral removal of the SCG delays the synchronization of feeding rhythms with a newly imposed diurnal lighting regimen, but, again, the response to pinealectomy was different (87). In fact, the elimination of pineal rhythmicity cannot account for all of the effects of SCGx on photic entrainment of feeding and locomotor activity rhythms. It can be suggested that SCGx alters the sympathetic innervation of hypothalamic structures implicated in the neural control of feeding, affecting the diurnal rhythm of food intake.

Rhythms in metabolism are orchestrated by the SCN and other inputs from different areas of the hypothalamus, like the mediobasal region, which plays a significant role in metabolic homeostasis (88–93). Other areas, like the dorsomedial hypothalamus, have an important role as a component of the SCN-independent food-entrainable oscillator (94–97). The circadian regulation of body weight depends on the integration of multiple signals of several hypothalamic areas, including the SCN, the arcuate nucleus, the ventromedial hypothalamic nucleus, and the paraventricular nucleus, that control appetite and food intake, deposition of fat, and energy expenditure (11, 53, 98). Melatonin not only couples circadian cues to many body functions but might also be a key player in the regulation of basal metabolic rate (99), independently of other SCG-innervated territories, such as the hypothalamus. In this sense, the results shown in this work provide evidence suggesting that SCGx may be affecting metabolism by changing the feeding pattern (i.e., increasing feeding during daytime), acting over peripheral clocks without affecting the SCN.

In conclusion, these findings provide insights into the metabolic and diurnal rhythms of ganglionectomized rats. SCGx is not only a good model to study the circadian clock influence on neuroendocrine functions, but a reliable approach to investigate the relationship between the circadian system and metabolism, as well as the role of the SCG innervation in the synchronization of the master circadian clock with the peripheral clocks, especially the ones that drive metabolic variables.

## Ethics Statement

All animal procedures were approved by the Institutional Animal Care and Use Committee at the School of Medicine, National University of Cuyo, Mendoza, Argentina (Protocol ID 9/2012) and were conducted in accordance with the National Institutes of Health’s Guide for Care and Use of Laboratory Animals and the Animal Research: Reporting *In Vivo* Experiments (ARRIVE) Guidelines.

## Author Contributions

MG and SP performed experiments for the paper; MF, MG, and SP analyzed data; MF, DG, EM, and SP wrote the manuscript; DG and EM provided reagents and funding for the study.

## Conflict of Interest Statement

The authors declare that the research was conducted in the absence of any commercial or financial relationships that could be construed as a potential conflict of interest.

## References

[1] Aschoff J. Human circadian rhythms in activity, body temperature and other functions. Life Sci Space Res (1967) 5:159–73. 11973844

[2] Stephan FK Zucker I. Circadian rhythms in drinking behavior and locomotor activity of rats are eliminated by hypothalamic lesions. Proc Natl Acad Sci U S A (1972) 69(6):1583–6. 10.1073/pnas.69.6.1583 4556464 PMC426753

[3] Van den Pol AN Powley T A fine-grained anatomical analysis of the role of the rat suprachiasmatic nucleus in circadian rhythms of feeding and drinking. Brain Res (1979) 160(2):307–26. 10.1016/0006-8993(79)90427-X 761068

[4] Rudic RD McNamara P Curtis AM Boston RC Panda S Hogenesch JB BMAL1 and CLOCK, two essential components of the circadian clock, are involved in glucose homeostasis. PLoS Biol (2004) 2(11):e377. 10.1371/journal.pbio.0020377 15523558 PMC524471

[5] Golombek DA Rosenstein RE. Physiology of circadian entrainment. Physiol Rev (2010) 90(3):1063–102. 10.1152/physrev.00009.2009 20664079

[6] Balsalobre A Brown SA Marcacci L Tronche F Kellendonk C Reichardt HM Resetting of circadian time in peripheral tissues by glucocorticoid signaling. Science (2000) 289(5488):2344–7. 10.1126/science.289.5488.2344 11009419

[7] Mohawk JA Green CB Takahashi JS. Central and peripheral circadian clocks in mammals. Annu Rev Neurosci (2012) 35:445–62. 10.1146/annurev-neuro-060909-153128 22483041 PMC3710582

[8] Pezuk P Mohawk JA Wang LA Menaker M. Glucocorticoids as entraining signals for peripheral circadian oscillators. Endocrinology (2012) 153(10):4775–83. 10.1210/en.2012-1486 22893723 PMC3512018

[9] Damiola F Le Minh N Preitner N Kornmann B Fleury-Olela F Schibler U. Restricted feeding uncouples circadian oscillators in peripheral tissues from the central pacemaker in the suprachiasmatic nucleus. Genes Dev (2000) 14(23):2950–61. 10.1101/gad.183500 11114885 PMC317100

[10] Challet E. Circadian clocks, food intake, and metabolism. Prog Mol Biol Transl Sci (2013) 119:105–35. 10.1016/B978-0-12-396971-2.00005-1 23899596

[11] Plano SA Casiraghi LP García Moro P Paladino N Golombek DA Chiesa JJ. Circadian and metabolic effects of light: implications in weight homeostasis and health. Front Neurol (2017) 8:558. 10.3389/fneur.2017.00558 29097992 PMC5653694

[12] Turek FW Joshu C Kohsaka A Lin E Ivanova G McDearmon E Obesity and metabolic syndrome in circadian Clock mutant mice. Science (2005) 308(5724):1043–5. 10.1126/science.1108750 15845877 PMC3764501

[13] Loudon AS Meng QJ Maywood ES Bechtold DA Boot-Handford RP Hastings MH. The biology of the circadian Ck1epsilon tau mutation in mice and Syrian hamsters: a tale of two species. Cold Spring Harb Symp Quant Biol (2007) 72:261–71. 10.1101/sqb.2007.72.073 18522517

[14] Scheer FA Hilton MF Mantzoros CS Shea SA. Adverse metabolic and cardiovascular consequences of circadian misalignment. Proc Natl Acad Sci U S A (2009) 106(11):4453–8. 10.1073/pnas.0808180106 19255424 PMC2657421

[15] Casiraghi LP Alzamendi A Giovambattista A Chiesa JJ Golombek DA. Effects of chronic forced circadian desynchronization on body weight and metabolism in male mice. Physiol Rep (2016) 4(8):e12743. 10.14814/phy2.12743 27125665 PMC4848717

[16] Pandi-Perumal SR Trakht I Srinivasan V Spence DW Maestroni GJ Zisapel N Physiological effects of melatonin: role of melatonin receptors and signal transduction pathways. Prog Neurobiol (2008) 85(3):335–53. 10.1016/j.pneurobio.2008.04.001 18571301

[17] Krauchi K Wirz-Justice A. Circadian clues to sleep onset mechanisms. Neuropsychopharmacology (2001) 25(5 Suppl):S92–6. 10.1016/S0893-133X(01)00315-3 11682282

[18] Nishida S Segawa T Murai I Nakagawa S. Long-term melatonin administration reduces hyperinsulinemia and improves the altered fatty-acid compositions in type 2 diabetic rats via the restoration of delta-5 desaturase activity. J Pineal Res (2002) 32(1):26–33. 10.1034/j.1600-079x.2002.10797.x 11841597

[19] Pandi-Perumal SR Zisapel N Srinivasan V Cardinali DP. Melatonin and sleep in aging population. Exp Gerontol (2005) 40(12):911–25. 10.1016/j.exger.2005.08.009 16183237

[20] Hussein MR Ahmed OG Hassan AF Ahmed MA. Intake of melatonin is associated with amelioration of physiological changes, both metabolic and morphological pathologies associated with obesity: an animal model. Int J Exp Pathol (2007) 88(1):19–29. 10.1111/j.1365-2613.2006.00512.x 17244335 PMC2517290

[21] Peschke E Bahr I Muhlbauer E. Melatonin and pancreatic islets: interrelationships between melatonin, insulin and glucagon. Int J Mol Sci (2013) 14(4):6981–7015. 10.3390/ijms14046981 23535335 PMC3645673

[22] Sun H Huang FF Qu S. Melatonin: a potential intervention for hepatic steatosis. Lipids Health Dis (2015) 14:75. 10.1186/s12944-015-0081-7 26199093 PMC4511016

[23] Zimmerman NH Menaker M. The pineal gland: a pacemaker within the circadian system of the house sparrow. Proc Natl Acad Sci U S A (1979) 76(2):999–1003. 10.1073/pnas.76.2.999 284425 PMC383119

[24] Klein DC Arylalkylamine N-acetyltransferase: “the Timezyme”. J Biol Chem (2007) 282(7):4233–7. 10.1074/jbc.R600036200 17164235

[25] Kumar V Gwinner E. Pinealectomy shortens resynchronisation times of house sparrow (Passer domesticus) circadian rhythms. Naturwissenschaften (2005) 92(9):419–22. 10.1007/s00114-005-0009-6 16151793

[26] Schwartz MD Wotus C Liu T Friesen WO Borjigin J Oda GA Dissociation of circadian and light inhibition of melatonin release through forced desynchronization in the rat. Proc Natl Acad Sci U S A (2009) 106(41):17540–5. 10.1073/pnas.0906382106 19805128 PMC2762670

[27] Touitou Y Reinberg A Touitou D. Association between light at night, melatonin secretion, sleep deprivation, and the internal clock: health impacts and mechanisms of circadian disruption. Life Sci (2017) 173:94–106. 10.1016/j.lfs.2017.02.008 28214594

[28] Bowers CW Dahm LM Zigmond RE. The number and distribution of sympathetic neurons that innervate the rat pineal gland. Neuroscience (1984) 13(1):87–96. 10.1016/0306-4522(84)90261-6 6493487

[29] Romeo HE Spinedi E Esquifino AI Estivariz F Cardinali DP. Anterograde nerve degeneration after superior cervical ganglionectomy coexists with a decrease in arginine vasopressin release in rats. Neuroendocrinology (1991) 54(4):346–52. 10.1159/000125912 1758576

[30] Cardinali DP Stern JE Peripheral neuroendocrinology of the cervical autonomic nervous system. Braz J Med Biol Res (1994) 27(3):573–99. 8081283

[31] Savastano LE Castro AE Fitt MR Rath MF Romeo HE Munoz EM. A standardized surgical technique for rat superior cervical ganglionectomy. J Neurosci Methods (2010) 192(1):22–33. 10.1016/j.jneumeth.2010.07.007 20637235

[32] Saboureau M Vivien-Roels B Pevet P. Pineal melatonin concentrations during day and night in the adult hedgehog: effect of a light pulse at night and superior cervical ganglionectomy. J Pineal Res (1991) 11(2):92–8. 10.1111/j.1600-079X.1991.tb00462.x 1757890

[33] Perreau-Lenz S Kalsbeek A Garidou ML Wortel J van der Vliet J van Heijningen C Suprachiasmatic control of melatonin synthesis in rats: inhibitory and stimulatory mechanisms. Eur J Neurosci (2003) 17(2):221–8. 10.1046/j.1460-9568.2003.02442.x 12542658

[34] Klein DC Weller JL Moore RY. Melatonin metabolism: neural regulation of pineal serotonin: acetyl coenzyme A N-acetyltransferase activity. Proc Natl Acad Sci U S A (1971) 68(12):3107–10. 10.1073/pnas.68.12.3107 4332009 PMC389600

[35] Siaud P Mekaouche M Maurel D Givalois L Ixart G Superior cervical ganglionectomy suppresses circadian corticotropic rhythms in male rats in the short term (5 days) and long term (10 days). Brain Res (1994) 652(2):273–8. 10.1016/0006-8993(94)90237-2 7953740

[36] Cardinali DP Vacas MI Gejman PV. The sympathetic superior cervical ganglia as peripheral neuroendocrine centers. J Neural Transm (1981) 52(1–2):1–21. 10.1007/BF01253092 7026734

[37] Gejman PV Cardinali DP Finkielman S Nahmod VE. Changes in drinking behavior caused by superior cervical ganglionectomy and pinealectomy in rats. J Auton Nerv Syst (1981) 4(3):249–59. 10.1016/0165-1838(81)90048-5 7299041

[38] Jallageas M Mas N Saboureau M Roussel JP Lacroix A. Effects of bilateral superior cervical ganglionectomy on thyroid and gonadal functions in the edible dormouse Glis glis. Comp Biochem Physiol Comp Physiol (1993) 104(2):299–304. 10.1016/0300-9629(93)90321-T 8095882

[39] Grkovic I Anderson CR. Calretinin-containing preganglionic nerve terminals in the rat superior cervical ganglion surround neurons projecting to the submandibular salivary gland. Brain Res (1995) 684(2):127–35. 10.1016/0006-8993(95)00392-4 7583213

[40] Castrillon PO Cardinali DP Pazo D Cutrera RA Esquifino AI. Effect of superior cervical ganglionectomy on 24-h variations in hormone secretion from the anterior hypophysis and in hypothalamic monoamine turnover during the preclinical phase of Freund’s adjuvant arthritis in rats. J Neuroendocrinol (2001) 13(3):288–95. 10.1046/j.1365-2826.2001.00627.x 11207944

[41] Ziegler KA Ahles A Wille T Kerler J Ramanujam D Engelhardt S Local sympathetic denervation attenuates myocardial inflammation and improves cardiac function after myocardial infarction in mice. Cardiovasc Res (2017) cvx227 10.1093/cvr/cvx227 PMC5852629 29186414

[42] Thone-Reineke C Kalk P Dorn M Klaus S Simon K Pfab T High-protein nutrition during pregnancy and lactation programs blood pressure, food efficiency, and body weight of the offspring in a sex-dependent manner. Am J Physiol Regul Integr Comp Physiol (2006) 291(4):R1025–30. 10.1152/ajpregu.00898.2005 16675628

[43] Garcia JB Romeo HE Basabe JC Cardinali DP. Effect of superior cervical ganglionectomy on insulin release by murine pancreas slices. J Auton Nerv Syst (1988) 22(2):159–65. 10.1016/0165-1838(88)90089-6 3288689

[44] Murlidhar Katira V Rameshbabu CS Mathur JS Saxena KK. Effect of pinealectomy on daily rhythm of blood glucose in rabbits. Indian J Exp Biol (1991) 29(3):278–9. 1874543

[45] Tan Y Ogawa H Effect of superior cervical sympathetic ganglionectomy on FSH, LH and GH cells of hypophyseal gland in female rats: a quantitative immunohistochemical study. Masui (1996) 45(10):1223–34. 8937018

[46] Dziedzic B Walczewska A. Gonadotropin-releasing hormone (GnRH) content in the median eminence after superior cervical ganglionectomy in ovariectomized and estrogen-treated rats. Exp Clin Endocrinol Diabetes (1997) 105(1):57–62. 10.1055/s-0029-1211728 9088896

[47] Cassaglia PA Griffiths RI Walker AM. Sympathetic nerve activity in the superior cervical ganglia increases in response to imposed increases in arterial pressure. Am J Physiol Regul Integr Comp Physiol (2008) 294(4):R1255–61. 10.1152/ajpregu.00332.2007 18216142

[48] Lindvall M Edvinsson L Owman C. Sympathetic nervous control of cerebrospinal fluid production from the choroid plexus. Science (1978) 201(4351):176–8. 10.1126/science.663649 663649

[49] Bjorklund A Owman C West KA Peripheral sympathetic innervation and serotonin cells in the habenular region of the rat brain. Z Zellforsch Mikrosk Anat (1972) 127(4):570–9. 10.1007/BF00306872 5045871

[50] Reiter RJ Hester RJ Interrelationships of the pineal gland, the superior cervical ganglia and the photoperiod in the regulation of the endocrine systems of hamsters. Endocrinology (1966) 79(6):1168–70. 10.1210/endo-79-6-1168 5951523

[51] Buttle HL. The effect of anterior cervical ganglionectomy on the seasonal variation in prolactin concentration in goats. Neuroendocrinology (1977) 23(2):121–8. 10.1159/000122660 895991

[52] Challet E Keeping circadian time with hormones. Diabetes Obes Metab (2015) 17(Suppl 1):76–83. 10.1111/dom.12516 26332971

[53] Buijs FN Leon-Mercado L Guzman-Ruiz M Guerrero-Vargas NN Romo-Nava F Buijs RM. The circadian system: a regulatory feedback network of periphery and brain. Physiology (Bethesda) (2016) 31(3):170–81. 10.1152/physiol.00037.2015 27053731

[54] Kalsbeek A Garidou ML Palm IF Van Der Vliet J Simonneaux V Pevet P Melatonin sees the light: blocking GABA-ergic transmission in the paraventricular nucleus induces daytime secretion of melatonin. Eur J Neurosci (2000) 12(9):3146–54. 10.1046/j.1460-9568.2000.00202.x 10998098

[55] Perreau-Lenz S Kalsbeek A Pevet P Buijs RM. Glutamatergic clock output stimulates melatonin synthesis at night. Eur J Neurosci (2004) 19(2):318–24. 10.1111/j.0953-816X.2003.03132.x 14725626

[56] Bartness TJ Wade GN. Photoperiodic control of seasonal body weight cycles in hamsters. Neurosci Biobehav Rev (1985) 9(4):599–612. 10.1016/0149-7634(85)90006-5 3909016

[57] la Fleur SE Kalsbeek A Wortel J van der Vliet J Buijs RM. Role for the pineal and melatonin in glucose homeostasis: pinealectomy increases night-time glucose concentrations. J Neuroendocrinol (2001) 13(12):1025–32. 10.1046/j.1365-2826.2001.00717.x 11722698

[58] Cipolla-Neto J Amaral FG Afeche SC Tan DX Reiter RJ. Melatonin, energy metabolism, and obesity: a review. J Pineal Res (2014) 56(4):371–81. 10.1111/jpi.12137 24654916

[59] Prunet-Marcassus B Desbazeille M Bros A Louche K Delagrange P Renard P Melatonin reduces body weight gain in Sprague Dawley rats with diet-induced obesity. Endocrinology (2003) 144(12):5347–52. 10.1210/en.2003-0693 12970162

[60] Szewczyk-Golec K Wozniak A Reiter RJ Inter-relationships of the chronobiotic, melatonin, with leptin and adiponectin: implications for obesity. J Pineal Res (2015) 59(3):277–91. 10.1111/jpi.12257 26103557

[61] Wolden-Hanson T Mitton DR McCants RL Yellon SM Wilkinson CW Matsumoto AM Daily melatonin administration to middle-aged male rats suppresses body weight, intraabdominal adiposity, and plasma leptin and insulin independent of food intake and total body fat. Endocrinology (2000) 141(2):487–97. 10.1210/endo.141.2.7311 10650927

[62] Cardinali DP Cano P Jimenez-Ortega V Esquifino AI. Melatonin and the metabolic syndrome: physiopathologic and therapeutical implications. Neuroendocrinology (2011) 93(3):133–42. 10.1159/000324699 21358175

[63] Buijs RM Escobar C Swaab DF. The circadian system and the balance of the autonomic nervous system. Handb Clin Neurol (2013) 117:173–91. 10.1016/B978-0-444-53491-0.00015-8 24095125

[64] Panda S Circadian physiology of metabolism. Science (2016) 354(6315):1008–15. 10.1126/science.aah4967 27885007 PMC7261592

[65] Bray MS Ratcliffe WF Grenett MH Brewer RA Gamble KL Young ME. Quantitative analysis of light-phase restricted feeding reveals metabolic dyssynchrony in mice. Int J Obes (Lond) (2013) 37(6):843–52. 10.1038/ijo.2012.137 22907695 PMC3505273

[66] Mukherji A Kobiita A Chambon P. Shifting the feeding of mice to the rest phase creates metabolic alterations, which, on their own, shift the peripheral circadian clocks by 12 hours. Proc Natl Acad Sci U S A (2015) 112(48):E6683–90. 10.1073/pnas.1519735112 26627259 PMC4672831

[67] Opperhuizen AL van Kerkhof LW Proper KI Rodenburg W Kalsbeek A. Rodent models to study the metabolic effects of shiftwork in humans. Front Pharmacol (2015) 6:50. 10.3389/fphar.2015.00050 25852554 PMC4371697

[68] Opperhuizen AL Wang D Foppen E Jansen R Boudzovitch-Surovtseva O de Vries J Feeding during the resting phase causes profound changes in physiology and desynchronization between liver and muscle rhythms of rats. Eur J Neurosci (2016) 44(10):2795–806. 10.1111/ejn.13377 27562056

[69] Yasumoto Y Hashimoto C Nakao R Yamazaki H Hiroyama H Nemoto T Short-term feeding at the wrong time is sufficient to desynchronize peripheral clocks and induce obesity with hyperphagia, physical inactivity and metabolic disorders in mice. Metabolism (2016) 65(5):714–27. 10.1016/j.metabol.2016.02.003 27085778

[70] Martin AI Lopez-Calderon A Tresguerres JA Gonzalez-Quijano MI Cardinali DP. Restraint-induced changes in serum luteinizing hormone, prolactin, growth hormone and corticosterone levels in rats: effect of superior cervical ganglionectomy. Neuroendocrinology (1995) 61(2):173–9. 10.1159/000126838 7753336

[71] Lee KE Youm JK Lee WJ Kang S Kim YJ. Polyphenol-rich apple extract inhibits dexamethasone-induced sebaceous lipids production by regulating SREBP1 expression. Exp Dermatol (2017) 26(10):958–60. 10.1111/exd.13319 28191675

[72] Wu T Yang L Jiang J Ni Y Zhu J Zheng X Chronic glucocorticoid treatment induced circadian clock disorder leads to lipid metabolism and gut microbiota alterations in rats. Life Sci (2017). 10.1016/j.lfs.2017.11.049 29196049

[73] Mahu I Domingos AI. The sympathetic neuro-adipose connection and the control of body weight. Exp Cell Res (2017) 360(1):27–30. 10.1016/j.yexcr.2017.03.047 28342901 PMC6616029

[74] Gilgen A Maickel RP Nikodijevic O Brodie BB Essential role of catecholamines in the mobilization of free fatty acids and glucose after exposure to cold. Life Sci (1962) 1:709–15. 10.1016/0024-3205(62)90138-8 13947863

[75] Bartness TJ Bamshad M. Innervation of mammalian white adipose tissue: implications for the regulation of total body fat. Am J Physiol (1998) 275(5 Pt 2):R1399–411. 9791054 10.1152/ajpregu.1998.275.5.R1399

[76] Rayner DV. The sympathetic nervous system in white adipose tissue regulation. Proc Nutr Soc (2001) 60(3):357–64. 10.1079/PNS2001101 11681810

[77] Zeng W Pirzgalska RM Pereira MM Kubasova N Barateiro A Seixas E Sympathetic neuro-adipose connections mediate leptin-driven lipolysis. Cell (2015) 163(1):84–94. 10.1016/j.cell.2015.08.055 26406372 PMC7617198

[78] Turtzo LC Marx R Lane MD. Cross-talk between sympathetic neurons and adipocytes in coculture. Proc Natl Acad Sci U S A (2001) 98(22):12385–90. 10.1073/pnas.231478898 11606782 PMC60063

[79] Kincl FA Chang CC Zbuzkova V Observation on the influence of changing photoperiod on spontaneous wheel-running activity of neonatally pinealectomized rats. Endocrinology (1970) 87(1):38–42. 10.1210/endo-87-1-38 5462978

[80] Quay WB Physiological significance of the pineal during adaptation to shifts in photoperiod. Physiol Behav (1970) 5(3):353–60. 10.1016/0031-9384(70)90110-1 5535786

[81] Quay WB Pineal homeostatic regulation of shifts in the circadian activity rhythm during maturation and aging. Trans N Y Acad Sci (1972) 34(3):239–54. 10.1111/j.2164-0947.1972.tb02679.x 4503408

[82] Finkelstein JS Baum FR Campbell CS Entrainment of the female hamster to reversed photoperiod: role of the pineal. Physiol Behav (1978) 21(1):105–11. 10.1016/0031-9384(78)90283-4 567813

[83] Redman JR Armstrong SM. Reentrainment of rat circadian activity rhythms: effects of melatonin. J Pineal Res (1988) 5(2):203–15. 10.1111/j.1600-079X.1988.tb00782.x 3367270

[84] Golombek DA Cardinali DP. Melatonin accelerates reentrainment after phase advance of the light-dark cycle in Syrian hamsters: antagonism by flumazenil. Chronobiol Int (1993) 10(6):435–41. 10.3109/07420529309059719 8111868

[85] Pitrosky B Kirsch R Malan A Mocaer E Pevet P. Organization of rat circadian rhythms during daily infusion of melatonin or S20098, a melatonin agonist. Am J Physiol (1999) 277(3 Pt 2):R812–28. 10484499 10.1152/ajpregu.1999.277.3.R812

[86] Carpentieri AR Pujolras MA Chiesa JJ Noguera AD Cambras T. Effect of melatonin and diazepam on the dissociated circadian rhythm in rats. J Pineal Res (2006) 40(4):318–25. 10.1111/j.1600-079X.2006.00320.x 16635019

[87] Baum MJ Light-synchronization of rat feeding rhythms following sympathectomy or pinealectomy. Physiol Behav (1970) 5(3):325–9. 10.1016/0031-9384(70)90105-8 5525712

[88] Bernardis LL Bellinger LL. The dorsomedial hypothalamic nucleus revisited: 1998 update. Proc Soc Exp Biol Med (1998) 218(4):284–306. 10.3181/00379727-218-44296 9714072

[89] Sahu A. Minireview: a hypothalamic role in energy balance with special emphasis on leptin. Endocrinology (2004) 145(6):2613–20. 10.1210/en.2004-0032 15044360

[90] Kalsbeek A Palm IF La Fleur SE Scheer FA Perreau-Lenz S Ruiter M SCN outputs and the hypothalamic balance of life. J Biol Rhythms (2006) 21(6):458–69. 10.1177/0748730406293854 17107936

[91] Sellix MT Egli M Poletini MO McKee DT Bosworth MD Fitch CA Anatomical and functional characterization of clock gene expression in neuroendocrine dopaminergic neurons. Am J Physiol Regul Integr Comp Physiol (2006) 290(5):R1309–23. 10.1152/ajpregu.00555.2005 16373438 PMC1457054

[92] Duncan MJ. Circannual prolactin rhythms: calendar-like timer revealed in the pituitary gland. Trends Endocrinol Metab (2007) 18(7):259–60. 10.1016/j.tem.2007.07.001 17689257

[93] Guilding C Hughes AT Brown TM Namvar S Piggins HD. A riot of rhythms: neuronal and glial circadian oscillators in the mediobasal hypothalamus. Mol Brain (2009) 2:28. 10.1186/1756-6606-2-28 19712475 PMC2745382

[94] Gooley JJ Schomer A Saper CB. The dorsomedial hypothalamic nucleus is critical for the expression of food-entrainable circadian rhythms. Nat Neurosci (2006) 9(3):398–407. 10.1038/nn1651 16491082

[95] Landry GJ Simon MM Webb IC Mistlberger RE. Persistence of a behavioral food-anticipatory circadian rhythm following dorsomedial hypothalamic ablation in rats. Am J Physiol Regul Integr Comp Physiol (2006) 290(6):R1527–34. 10.1152/ajpregu.00874.2005 16424080

[96] Landry GJ Yamakawa GR Webb IC Mear RJ Mistlberger RE. The dorsomedial hypothalamic nucleus is not necessary for the expression of circadian food-anticipatory activity in rats. J Biol Rhythms (2007) 22(6):467–78. 10.1177/0748730407307804 18057321

[97] Moriya T Aida R Kudo T Akiyama M Doi M Hayasaka N The dorsomedial hypothalamic nucleus is not necessary for food-anticipatory circadian rhythms of behavior, temperature or clock gene expression in mice. Eur J Neurosci (2009) 29(7):1447–60. 10.1111/j.1460-9568.2009.06697.x 19519629

[98] Morton GJ Cummings DE Baskin DG Barsh GS Schwartz MW. Central nervous system control of food intake and body weight. Nature (2006) 443(7109):289–95. 10.1038/nature05026 16988703

[99] Owino S Contreras-Alcantara S Baba K Tosini G. Melatonin signaling controls the daily rhythm in blood glucose levels independent of peripheral clocks. PLoS One (2016) 11(1):e0148214. 10.1371/journal.pone.0148214 26824606 PMC4732609

